# Investigation of dosimetric differences between the TMR 10 and convolution algorithm for Gamma Knife stereotactic radiosurgery

**DOI:** 10.1120/jacmp.v17i6.6347

**Published:** 2016-11-08

**Authors:** Alvaro Rojas‐Villabona, Neil Kitchen, Ian Paddick

**Affiliations:** ^1^ Department of Neurosurgery National Hospital for Neurology and Neurosurgery London UK; ^2^ The Gamma Knife Centre at Queen Square National Hospital for Neurology and Neurosurgery London UK

**Keywords:** Gamma Knife, radiosurgery, dose planning, convolution, tissue maximum ratio algorithm

## Abstract

Since its inception, doses applied using Gamma Knife Radiosurgery (GKR) have been calculated using a simple TMR algorithm, which assumes the patient's head is of even density, the same as water. This results in a significant approximation of the dose delivered by the Gamma Knife. We investigated how GKR dose calculations varied when using a new convolution algorithm clinically available for GKR planning that takes into account density variations in the head compared with the established calculation algorithm. Fifty‐five patients undergoing GKR and harboring 85 lesions were voluntarily and prospectively enrolled into the study. Their clinical treatment plans were created and delivered using TMR 10, but were then recalculated using the density correction algorithm. Dosimetric differences between the planning algorithms were noted. Beam on time (BOT), which is directly proportional to dose, was the main value investigated. Changes of mean and maximum dose to organs at risk (OAR) were also assessed. Phantom studies were performed to investigate the effect of frame and pin materials on dose calculation using the convolution algorithm. Convolution yielded a mean increase in BOT of 7.4% (3.6%–11.6%). However, approximately 1.5% of this amount was due to the head contour being derived from the CT scans, as opposed to measurements using the Skull Scaling Instrument with TMR. Dose to the cochlea calculated with the convolution algorithm was approximately 7% lower than with the TMR 10 algorithm. No significant difference in relative dose distribution was noted and CT artifact typically caused by the stereotactic frame, glue embolization material or different fixation pin materials did not systematically affect convolution isodoses. Nonetheless, substantial error was introduced to the convolution calculation in one target located exactly in the area of major CT artifact caused by a fixation pin. Inhomogeneity correction using the convolution algorithm results in a considerable, but consistent, dose shift compared to the TMR 10 algorithm traditionally used for GKR. A reduction of the prescription dose may be necessary to obtain the same clinical effect with the convolution algorithm. Head shape definition using CT outlining can reduce treatment uncertainty from head shape approximations.

PACS number(s): 87.53.‐j; 87.55.D; 87.55.kd

## I. INTRODUCTION

Gamma Knife radiosurgery (GKR) relies on mathematical algorithms to predict the distribution of ionizing radiation in the brain.^(1)^ The dose distribution is affected by electron density heterogeneities of the tissues and this is a well‐established concept that is compensated for in conventional radiotherapy and other forms of radiosurgery.^(2)^ However, for a number of reasons heterogeneity corrections have been unavailable for GKR. Doses applied with GKR have been traditionally calculated using a simpler water‐based algorithm.^(3)^ The tissue maximum ratio (TMR 10) is the most recent enhancement of the water‐based dose calculation algorithm used for GKR and it relies on a number of approximations to enable fast isodose computation during treatment planning. One of the most significant of these is the approximation of the head to water‐equivalent density, and this could introduce important uncertainty to isodose and beam‐on time calculations due to the increased electron density of brain and bone (relative to water) and the near‐zero density of air cavities in the skull.^(4)^

The TMR 10 algorithm requires input data of off‐axis ratios (dose profiles) and other parameters, such as output factors, attenuation/virtual attenuation coefficients, virtual source‐to‐focus distances, and scaling distances. The data used as input for the simulation have been extracted by analyzing Monte Carlo simulations and subsequently adapting the calculation model to the results.^(5)^ This simple algorithm was a practical method to overcome the relatively slow processing capabilities of older workstations, but with the advent of faster processors, the effect of tissue inhomogeneities can finally be calculated in reasonable time during the treatment planning process.^(6)^

The ability to account for tissue heterogeneity in GKR has become available in the form of a convolution algorithm.^(7)^ It calculates dose by convolving a field describing the total amount of energy released by primary photons per unit mass (TERMA) with kernels describing how the energy is distributed by secondary particles.^(2)^ To account for tissue heterogeneities in the head, the TERMA and the kernels are scaled by material densities obtained from computed tomography (CT) Hounsfield units, which are directly proportional to the electron density of the tissues.^(1)^

The convolution algorithm is known to more accurately predict dose distributions across the brain.^8^, ^9^ However, doses used for GKR were tested and optimized using water‐based algorithms over the last few decades and the dosimetric differences between the water‐based and convolution algorithms need to be better understood before this method can be confidently employed in a clinical setting. This study is aimed to understand the dosimetric implications of using convolution algorithm for GKR.

## II. MATERIALS AND METHODS

Treatment plans of a representative group of patients were created using the TMR 10 algorithm and replanned using the convolution algorithm. Beam‐on‐time, which is proportional to treatment dose, and a number of metrics commonly used to evaluate dose distribution, such as the Paddick Conformity Index (PCI), gradient index (GI), and coverage, were estimated with both algorithms. Changes of mean and maximum dose to organs at risk (OAR) were also assessed. Phantom studies were performed to investigate the effect of frame and pin materials on dose calculation using the convolution algorithm.

### A. Patients

Fifty‐five patients undergoing GKR for a variety of intracranial diseases between September 2013 and June 2014 were recruited for the study. Table 1 shows the demographic and diagnosis details of these subjects. The study was approved by the Research Ethics Committee, which is the UK equivalent to an Institutional Review Board (IRB). Written consent was received from all participants for an additional stereotactic CT scan of the head which is not part of the standard imaging procedure for planning of GKR in our center.

**Table 1:** Demographic and diagnosis details of the study subjects

*Age Mean (range): 53.4 (26–76) Female: 32 (58.2%)*		
*Diagnosis*	*Patients*	*Targets (%)*
Meningioma	16	24 (28.2)
Acoustic neuroma	17	17 (20.0)
AVM	11	12 (14.2)
Trigeminal neuralgia	4	4 (4.7)
Multiple metastases	3	24 (28.2)
Single metastases	2	2 (2.4)
Paraganglioma	2	2 (2.4)
Total	55	85 (100%)

AVM = arteriovenous malformation.

### B. Radiosurgery planning procedure with TMR 10

**Figure 1:**
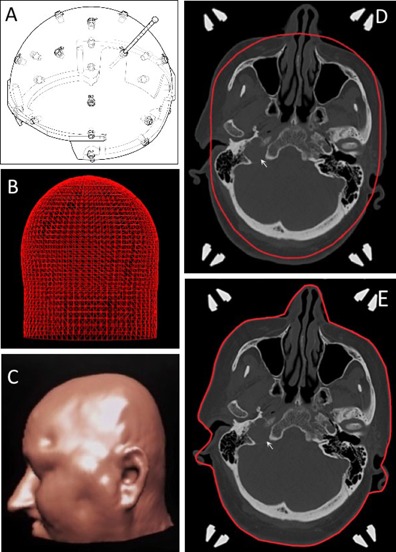
Head shape approximation methods. Skull scaling instrument (a) and the 3D model (b) generated with 24 manual measurements of the patient's head. Segmentation of the head surface using CT outlining produces a more accurate head shape model (c) and visual assessment of the CT scans can easily reveal discrepancies between the manual method (d) and CT outlining (e) in a subject with a right paraganglioma (arrow).

A Leksell stereotactic coordinate frame G (Elekta Instruments AB, Stockholm, Sweden) was applied to the head of the patients using titanium pins. Twenty‐four manual measurements of the patient's head were manually taken for head shape approximation using the skull scaling instrument or “bubble” method shown in Fig. 1(a).

Stereotactic imaging for planning included three‐dimensional (3D) postcontrast T1 and T2 weighted sequences acquired with a Magnetom Avanto 1.5T MRI system (Siemens AG, Erlangen, Germany) as follows: T1 weighted: Fast Low Angle SHot (FLASH); T2 weighted: Constructive Interference in Steady State (CISS); acquisition matrix: 448×448; slice thickness: 1.5 mm, no overlap; FoV: 210×210mm; voxel size: 0.47×0.47×1.5mm. GKR treatment plans were created using Leksell GammaPlan 10.1 and the water‐based TMR 10 algorithm (Elekta Instrument AB). Targets and OAR were delineated and a treatment plan produced of up to several radiation isocenters to conformaly cover the target volume.^(10)^ Dose and prescription isodose were chosen based on recognized standards for each pathology.^(11)^ Treatments were delivered using a Leksell Gamma Knife Perfexion (Elekta AB).

The quality of head shape approximation with the scaling instrument and target position were thought to influence potential differences of beam‐on time (BOT) between the dose calculation algorithms and they were, therefore, further assessed for each target. Discrepancies between the head outline obtained from manual measurements (red line, Fig. 1(d)) and the head contour as observed in the CT scan were manually assessed by a single observer (ARV) at the axial level of the target initially. Multiplane evaluations were subsequently performed, looking for discrepancies above or at the level of the target, which is the expected trajectory of the beams. The maximum distance between the actual head contour and the line of the head shape from manual measurements were recorded using arbitrary ranges as follows: less than 0.5 cm, 0.5 to 1 cm, and more than 1 cm. The position of the target in the head was evaluated with reference to the skull base, the head surface and the apex, manually measuring the minimum distance between these structures and the margin of the target. The targets were then classified using arbitrary thresholds — that is to say, skull base lesion if less than 2.5 cm from any bony structure on the base of the skull (n: 35), apex target if less than 2.5 cm from the highest point of the head in the stereotactic system (n: 10), and superficial if less than 2.5 cm from the head surface at any point (n: 28). These categories were not mutually exclusive.

### C. CT imaging and electron density calibration

Stereotactic noncontrast CT scanning of the whole head was performed in all subjects using a Siemens Somatom Definition AS multislice helical CT scanner (Siemens AG, Forchheim, Germany). Acquisition matrix: 512 x 512; slice thickness: 1.5 mm, no overlap; FoV: 240×240mm; voxel size: 0.47×0.47×1.5mm. A frame‐CT adapter was used to position the patient on the CT scanner.

A Gammex 467 tissue characterization phantom (Gammex. Middelton, WI) together with the CT scanner and parameters above were used to establish the relationship between electron density ($\rho_{e}$) of various tissues and their corresponding CT number in Hounsfield units (HU) for that specific scanner.^(12)^ The CT number and electron density relative to water of the rod materials for the phantom used were entered in the treatment planning system and used as a reference for inhomogeneity corrections using the convolution algorithm.

### D. Treatment replanning with convolution algorithm

The original treatment plans calculated with the TMR 10 algorithm were transferred to an independent Leksell GammaPlan 10.1 workstation, calibrated with the same reference dose (3.484 Gy/min to 20.10.2012), for replanning. The head segmenting tool in Leksell GammaPlan 10.1 was used to generate a 3D model of the patient's head from CT images (Figs. 1(c) and 1(e)). Minor modifications were performed manually to correct errors in irregular areas such as nose and ears and to completely exclude the stereotactic frame which can be mistakenly included in the model.

The treatment plan was initially recalculated with the same TMR 10 algorithm, but using the new head shape obtained from CT outlining. All other treatment parameters including prescription dose, percentage isodose, number and location of isocenters, and collimator size remained locked. Under these conditions, it can be safely assumed that BOT is directly proportional to delivered dose.

Electron density was subsequently calculated for each case using the CT scans and parameters from the calibration procedure above. The CT fiducial indicator box was excluded from the electron density calculations in GammaPlan and the treatment plans re‐calculated using the convolution algorithm. Dose calculations in Leksell GammaPlan 10.1 can be made independently for each individual target or summed to account for scatter from other targets in the case of multiple lesions. The latter method better represents the dose delivered to the patient and this was used to obtain the study figures.

### E. Statistical analysis

The nonparametric Wilcoxon signed‐rank test was primarily used to assess the significance of differences in BOT between the treatment plans. The actual difference, in minutes, between treatment plans does not fully describe the effect of heterogeneity correction for GKR planning. Therefore, percentage difference in BOT was calculated for each target and used for further statistical analysis. BOT percentage difference is normally distributed and the independent samples *t*‐test was used to compare target groups (e.g., skull base vs. nonskull base targets). Analysis of variance (ANOVA) was used to compare percentage difference in BOT between diagnoses and Spearman's rank correlation coefficient (ρ) was used to assess the relationship between percentage difference in BOT and other numerical variables (i.e., TV, number of isocenters, BOT) with the convolution algorithm. Each target was considered an independent study element for statistical purposes and data analysis was performed using the Statistical Package for the Social Sciences (SPSS) software (IBM SPSS Statistics for Windows, Version 22.0. IBM Corp. Released 2013. Armonk, NY: IBM Corp).

### F. Effect of frame and pin materials on convolution calculation

The effect of CT artifact from the frame materials on inhomogeneity correction with the convolution algorithm was evaluated using a Leksell Gamma Knife Solid Water dosimetry phantom (Elekta Instruments AB). The phantom was initially scanned without the Leksell G frame fixation posts and pins using the same CT scanner and parameters above. Subsequently, the frame fixation posts and titanium pins were added to the phantom setup and scanned under the same conditions. The angled long insulated posts (155 mm) were used anteriorly and the medium straight posts (110 mm) posteriorly, to mimic a typical clinical setup. Posts were positioned in the Z (sup‐inf) direction to maximize the chance that some beams passing through the calibration point (100,100,100) would pass through the pins. Typical clinical pin lengths of 35 mm anteriorly and 45 mm posteriorly were used.

The CT fiducial indicator box was then added to the phantom arrangement which was rescanned using different pin materials (i.e., titanium, aluminum, and older style aluminium/tungsten carbide tip pins). An experimental GKR plan was generated using the convolution algorithm and a single 4 mm shot located in the center of the coordinate system (100,100,100). The maximum dose was set to 100 Gy and the dose rate was 2.704 Gy on the day of the experiment. Convolution plans were then calculated using the CT scans from each of the scenarios described above. The planning procedure was also performed with single isocenters of the 8 and 16 mm collimators.

## III. RESULTS

In total 85 targets were treated in 55 subjects recruited for the study. These were adequately distributed across the head with 41% of the targets located less than 2.5 cm from a bony structure in the skull base and 12% of the targets located less than 2.5 cm from the apex. Meningiomas (24 targets in 16 patients) and brain metastases (26 lesions in 5 patients) were the most common lesions comprising around two‐thirds of the study targets (Table 1). Four AV M patients had undergone partial embolization of their vascular lesion with 25%–50% Glubran (N‐butyl‐cyanoacrylate and metacrylossisulfolane; GEM srl, Viareggio, Italy) suspended in ethiodized oil. Table 2 summarizes the estimates of BOT, coverage, PCI, and GI for the three treatment plans produced per target: A. TMR 10 algorithm and head definition from manual measurements, B. TMR 10 algorithm and head definition from CT scans, and C. Convolution algorithm and head definition from CT scans.

**Figure 2:**
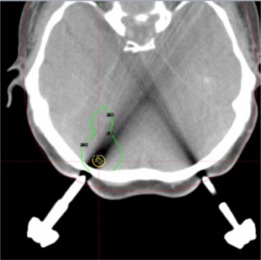
CT artifact from titanium fixation pin introducing significant error to the convolution calculation. Small brain metastasis (TV: 0.057 ml) located precisely under the Leksell G frame pin causes significant CT scan distortion and shorter BOT if the convolution algorithm is used.

BOT calculated with the convolution algorithm was longer for all the study targets, except a very small metastatic lesion which was located precisely under the frame fixation pin, as shown in Fig. 2. The CT artifact generated by the titanium pin introduced significant error to the convolution calculation through an abnormally low‐density artifact in the CT scan (HU: ‐664.4,SD:65.84; $\rho_{e}:0.291$, < water). This resulted in a shorter BOT if the convolution algorithm was used compared to TMR 10 (6.08 vs. 6.10 min, respectively). This lesion was excluded from further analysis. The percentage difference in BOT between treatment plans, for the 84 targets included in the analysis, are summarized in Table 3. No significant difference in coverage, PCI, or GI was observed between the head shape definition methods or dose algorithms.

**Table 2:** GKR plans calculated with different dose calculation algorithms and head shape approximation method. Parameters of treatment plans created using the TMR 10 algorithm and head approximation with the skull scaling instrument (A) and recalculated using head definition from CT scan outlining (B) and the convolution algorithm (C). PCI = Paddick conformity index, GI = gradient index

*Parameter*	*A. TMR 10 + manual measurements mean (min ‐ max) median; SD*	*B. TMR 10 + CT head definition mean (min ‐ max) median; SD*	*C. Convolution + CT head definition mean (min ‐ max) median; SD*
Beam‐on time (min)	31.12 (6.5 – 83.9)	31.59 (6.6 – 85.5)	33.39 (6.8 – 89.3)
n: 84^(a)^	30.2; 18.6	30.7; 18.9	32.7; 19.9
Coverage (%)	97.5 (94.3 – 100)	97.4 (94.3 – 100)	97.0 (91 – 100)
n: 80^(a,b)^	97.0; 1.58	97.0; 1.6	96.6; 0.83
PCI	0.82 (0.48 – 0.93)	0.82 (0.48 – 0.93)	0.82 (0.51 – 0.93)
n: 52^(a,b,c)^	0.84; 0.08	0.84; 0.08	0.84; 0.08
GI	2.776 (2.48 – 3.52)	2.776 (2.48 – 3.52)	2.749 (2.46 – 3.53)
n: 45^(a,c,d)^	2.730; 0.244	2.730; 0.245	2.660; 0.253

AV M = arteriovenous malformation.

a 1 target excluded due to its location in the area of pin distortion in the CT scan.

b No treatment volume calculated for trigeminal neuralgia cases

c PCI and GI were ignored for 28 small lesions with $\text{TV}<0.5\text{cc}.^{\left(20,21\right)}$

d GI was not calculated for 11 lesions with close proximity to another target.

**Table 3:** Change in BOT between treatment plans calculated with different head shape approximation methods and dose calculation algorithms

	*Manual Measurements vs. CT Head Definition mean (min ‐ max) STD; (p)* ^(b)^	*TMR 10 vs. Convolution mean (min ‐ max) STD; (p)* ^(b)^	*Overall Difference mean (min ‐ max) STD; (p)* ^(b)^
Beam‐on time % difference n: 84^a^	1.45% (0.0 – 3.4) 0.76; (<0.001)	5.86% (2.1 – 8.8) 1.21; (<0.001)	7.39% (3.6 – 11.6) 1.42; (<0.001)

a 1 target excluded; very small lesion in the area of the pin artifact.

b Wilcoxon signed‐rank test.

### A. Dosimetric effect of head approximation

Head shape definition using CT outlining resulted in an average increase of 1.45% (95%CI:1.3‐1.6; p<0.001) in BOT compared to treatment plans using manual measurements with the skull scaling instrument. BOT with CT outlining was the same (n: 8) or longer in all cases and the maximum difference observed was 3.4%. The latter was a skull base target with significant discrepancies between the head approximation methods (Figs. 1(d) and 1(e)). Visual evaluation of the head shape generated from manual measurements showed a discrepancy of more than 0.5 cm at the axial level of 70% of the targets and a multiplane discrepancy of more than 1 cm was observed above or at the level of 31% of the targets. The change in BOT for lesions with a discrepancy of more than 0.5 cm at the axial level of the target (1.7%; 95% CI: 1.5–1.9) was significantly higher than the rest of the lesions (0.9%; 95% CI: 0.6–1.2); p>0.001. Similarly, lesions with more than 1 cm discrepancy above or at the level of the target (1.6%; 95% CI: 1.4–1.7) had significantly higher changes in BOT compared to targets with less than 0.5 cm multiplane discrepancy (0.6%; 95% CI: 0.3–0.9); p>0.001.

### B. Dosimetric effect of convolution algorithm

GKR treatment plans calculated using the convolution algorithm were on average 5.9% longer in BOT than the TMR 10 plans delivered to the patients. That is, if convolution algorithm were used to treat this group of subjects, the dose delivered to them would be 5.9% higher. This is a statistically significant difference ranging from 2.1% to as much as 8.8%. The overall change in BOT resulting from the use of convolution algorithm combined with head definition from CT scans was 7.4% on average with a maximum observed of 11.6%.

Table 4 shows the relative difference in BOT between the planning methods per diagnosis. Convolution seems to reveal a fairly consistent shift from TMR 10 for indications where the target location is the same (e.g., vestibular schwannoma). For indications where the location varies widely (e.g., meningiomas or metastases) convolution seems to demonstrate greater dosimetric inconsistencies. The analysis of variance (ANOVA), however, failed to demonstrate a significant difference in BOT percentage between the diagnoses (p=0.115).

Location of the target in the head, specifically the depth, appears to negatively correlate with change in BOT between the TMR 10 and convolution algorithm. Tumors located less than 2.5 cm from the surface at any point in the head showed greater changes in BOT compared to deeper lesions, 5.5% (95% CI: 5.2–5.8); SD: 1.17 vs. 6.5% (95% CI: 6.1–6.9); 1.03, p<0.115, respectively. Similarly, distance from the edge of the target to the head surface negatively correlate with percentage change in BOT between the algorithms (ρ:‐0.36,p=0.001).

Change in BOT for targets located in the skull base was not significantly different from those distant to that bony structure (5.6% vs. 6.0%; p=0.1). Similarly, no relative difference was noted between targets located in the apex and the rest of the lesions distributed across the head anatomy (6.2% vs. 5.8%; p=0.27). BOT percentage difference between the planning algorithms did not correlate with target volume (ρ: 0.12;p=0.2), number of shots (ρ:−0.005;p=0.96), BOT with TMR 10 algorithm (ρ:‐0.14;p=0.19) or difference in BOT between the head shape definition methods (ρ:0.08;p=0.47). BOT difference in AVM patients who had undergone partial glue embolization (5.2%; 95% CI: 4.3–6.1; SD: 0.56) was comparable to patients without previous endovascular treatment (5.0%; 95% CI: 4.2–5.7; SD: 0.8), and no significant difference between them was noted (p=0.6).

**Table 4:** Relative difference in BOT between the TMR 10 and convolution algorithm per diagnosis

	*TMR 10 vs. Convolution mean (min ‐ max) STD; (p)* ^(b)^	*Overall Difference Convolution + head definition from CT scans mean (min ‐ max) STD; (p)* ^(b)^
AVM n:12	5.1% (3.7 – 6.3) 0.76; (0.002)	6.0% (4.7 – 8.0) 1.0; (0.002)
Metastases n:25^(a)^	5.8% (2.0 – 7.9) 1.42; (<0.001)	7.1% (3.6 – 10.1) 1.63; (<0.001)
Meningioma n:24	6.2% (4.4 – 8.6) 1.03; (<0.001)	7.8% (6.0 – 11.6) 1.24; (<0.001)
Trigeminal neuralgia n:4	5.4% (4.7 – 6.8) 0.95; (0.068)	7.3% (6.5 – 8.3) 0.74; (0.068)
Acoustic neuroma n:17	5.9% (4.5 – 7.6) 0.94; (<0.001)	7.9% (6.6 – 9.8) 0.85; (<0.001)
Paraganglioma n:2	6.2% (3.5 – 8.8) 3.75; (0.18)	8.7% (7.0 – 10.4) 2.35; (0.18)

a 1 target excluded due to its location in the area of pin distortion in the CT scan.

b Wilcoxon signed‐rank test.

### C. Dose to organs at risk

Clinically relevant OARs were defined in 28 subjects and differences in mean and maximum dose between the TMR 10 and convolution plans were calculated. The ipsilateral cochlea was the OAR in 24 cases with vestibular schwannoma, cerebellopontine angle meningioma, paraganglioma, and trigeminal neuralgia. The mean and maximum calculated dose to the cochlea with the convolution algorithm was approximately 7% lower than equivalent estimates obtained with the TMR 10 algorithm, as shown in Table 5. The optic apparatus was in close proximity to the target in four subjects and a lesser effect on mean and maximum dose to this OAR (2.0% and 2.4%, respectively) was noted between the planning algorithms.

**Table 5:** Difference in dose to organs between the TMR 10 and convolution algorithm

*Organs at Risk*		*TMR 10 mean (95%CI); STD*	*Convolution mean (95%CI); STD*	*% Difference mean (min ‐ max); STD*
Cochlea	mean dose (Gy)	2.7 (2.2–3.2);1.1	2.5 (2.1–3.0);1.0	‐7.3% (3.6–11.1);2.12
n: 24	max dose (Gy)	4.8 (3.6–5.9);2.7	4.5 (3.3–5.6);2.7	‐7.0% (1.6–11.7);2.49
Optic apparatus	mean dose (Gy)	3.5 (2.3–4.7);0.8	3.4 (2.2–4.5);0.7	‐2.0% (0.0–3.1);1.4
n: 4	max dose (Gy)	6.5 (4.9–8.2);1.05	6.4 (4.6–8.2);1.1	‐2.4% (1.3–5.3);1.89

### D. The effect of frame and pin materials on convolution calculation

The effect of CT distortion from the stereotactic frame on the convolution algorithm was assessed by means of change in BOT if an identical treatment plan was calculated using CT scans acquired with and without the Leksell G frame. The experiment demonstrated a maximum 4% longer BOT using the CT acquired with the posts and pins for the plan composed of a single shot of the 4 mm collimator. This effect was smaller for a similar plan with the 8 mm collimator and no effect at all was seen for the 16 mm collimator (Table 6).

No significant difference in BOT was seen if the titanium or aluminum pins were used for single shot plans of the 4, 8, and 16 mm. Only a small change of the order of 0.8% was seen for the 4 mm collimator plan if the aluminum/tungsten carbide tip pins were used compared to titanium or aluminum pins.

**Table 6:** Effect of the stereotactic frame on the convolution algorithm

	*Phantom Only BOT, minutes*	*Frame and Pins No Fiducial Indicator Box BOT, minutes*	*Percentage Difference*
4 mm	73.23	76.21	4.0 %
8 mm	43.79	44.56	1.7 %
16 mm	37.34	37.26	‐0.2 %

## IV. DISCUSSION

This study aimed to evaluate the dosimetric implications of using inhomogeneity corrections with the convolution algorithm for GKR. The novel algorithm, available in the GKR planning system, was compared to TMR 10 which is the standard water‐based algorithm traditionally used in GKR. Fifty‐five actual GKR treatment plans were recalculated with the convolution algorithm, keeping all other treatment parameters unchanged, and the study provided clinically relevant information on the magnitude of dose approximations traditionally accepted with the TMR 10 algorithm. The overall dose difference if convolution algorithm is used along with head definition from CT outlining is 7.4%, on average, and the maximum observed was 11.6%.

However, 1.5% of this amount is due to the increased accuracy of the head contour from the CT scans, as opposed to manual measurements from the skull scaling instrument.

### A. Dose implications of convolution algorithm for GKR

Doses currently used for GKR are the result of several decades of empirical optimization using water‐based algorithms. This titration process has resulted in a set of dosage recommendations shown to provide maximum clinical efficacy with the lowest morbidity.^(11)^ These doses have incorporated the uncertainty inherent to water‐based algorithms, and treatment plans calculated with the new calculation algorithm should therefore be adequately understood and adjusted to ensure the dose delivered is comparable.

The increment in dose to the target, attributable to inhomogeneity corrections, if exactly the same TMR 10 treatment plan is recalculated with the convolution algorithm was found to be 5.9% in our study. Similar results have been reported with phantom and clinical experiments.^(9)^ Xu et al.^(8)^ reported an average dose difference of 6.5% between the convolution and the TMR classic algorithm using a single shot placed in different positions along the x‐, y‐, and z‐axes on the stereotactic system with varying collimator sizes in a polystyrene phantom and a human head CT scan. Their study compared the dose calculation algorithms with a fixed geometry and established the baseline performance of the convolution algorithm. Similarly, Nakazawa et al.^(4)^ found a 1%–7% change of absolute dose to the target in 29 cases of vestibular schwannomas which were replanned with the convolution algorithm. Our study does not only quantify the uncertainty of the water‐based algorithm in a larger group of patients with different intracranial conditions but it also informs radiosurgery prescribers on dose adjustments that may be required if the convolution algorithm is to be used clinically. For example, in a typical trigeminal neuralgia case a maximum dose of 80 Gy is planned with the TMR 10 algorithm. Replanning this treatment with the convolution algorithm (assuming that homogeneity correction better simulates dose distribution) reveals that this target would actually receive a lower dose of around 76 Gy when treated with TMR 10. Table 7 shows similar estimates for other pathologies and demonstrates the rather conservative approach of the water‐based algorithm where the uncertainty always results in “undertreating” the target. However, if the trigeminal neuralgia patient above was to be treated with the convolution algorithm, 80 Gy would actually be delivered to the target and this would indeed exceed the originally intended dose.

The clinical significance of dose differences between the TMR 10 and convolution algorithm is debatable and it was out of the scope of this study. No clinical studies have been published reporting outcomes of patients treated with the convolution algorithm, but evidence from standard radiotherapy suggests that doses should be adjusted to obtain the same clinical effect if a homogeneity correction is to be used.^13^, ^14^ The dose shift in our study seems to be consistent, particularly for tumors with the same location, and a simple dose reduction could potentially be sufficient to compensate for the differences between the planning algorithms (Table 7). Phantom‐based studies initially demonstrated substantial changes in dose distribution in bone tissue and tissue interfaces.^(15)^ However, no substantial difference in dose distribution surrogates, such as gradient index, PCI, and coverage, was noted in our study. Nakasawa et al.^(4)^ also reported no change of relative dose distribution by visual assessment of the plans and suggested that the setting of multiple beams from all directions would offset the discrepancy of the dose distribution around the target. The latter could also explain our finding of lower difference in BOT for targets deeply located in the brain where a higher degree of uncertainty compensation takes place as the beams travel through different tissue densities.

**Table 7:** Dosimetric differences between the TMR 10 and convolution algorithm for GKR per diagnosis

	*Prescription Dose*	*BOT % Difference TMR 10 vs. Convolution mean (min ‐ max)*	*Dosimetric Change With Convolution (Gy)*
AV M n:12	25 Gy	5.1% (3.7 – 6.3)	1.27 Gy (0.9 – 1.6)
Metastases n:25	25 Gy	5.8% (2.0 – 7.9)	1.45 Gy (0.5 – 1.9)
Meningioma n:24	15 Gy	6.2% (4.4 – 8.6)	0.93 Gy (0.7 – 1.3)
Trigeminal neuralgia n:4	80 Gy	5.4% (4.7 – 6.8)	4.32 Gy (3.7 – 5.4)
Acoustic neuroma n:17	13 Gy	5.9% (4.5 – 7.6)	0.76 Gy (0.5 – 1.0)
Paraganglioma n:2	15 Gy	6.2% (3.5 – 8.8)	Gy (0.5 – 1.3)

The dosimetric differences between the planning algorithms in our study were relatively consistent particularly for targets located in the same area (i.e., trigeminal neuralgia and vestibular schwannomas) and keeping the same absolute dose through a simple dose reduction if the convolution algorithm is used may be more relevant for these pathologies. The dose uncertainty with TMR 10, however, seems to be less predictable for pathologies with variable location in the brain, such as metastases or meningiomas, and the convolution algorithm could better simulate the true dose delivered to these individual targets. Further clinical studies and close monitoring of outcomes for patients treated with the convolution algorithm may be prospectively conducted to investigate potential differences on efficacy and side effects profile. A further implication of using a new dose calculation algorithm for GKR is the potentially poor comparability between clinical studies performed using the TMR 10 and the convolution algorithm in the future.

Our study also investigated differences in dose to OAR with the convolution algorithm, particularly the cochlear apparatus. Our findings demonstrate that doses delivered to the cochlea with the TMR 10 algorithm are actually 7.0% lower than initially thought. This difference is well explained by the high density of the temporal bone where the cochlea is embedded and reflects the fact that TMR 10 does not take into account attenuation of the beams as they travel through different tissue densities. A further degree of reduction in dose to the cochlea could take place if the prescription dose with the convolution algorithm were to be adjusted to deliver the same dose traditionally delivered to the targets with the TMR 10 algorithm.

### B. Potential dose calculation inaccuracies with convolution

The convolution algorithm is by definition a better method to predict dose distribution in the brain, and most modern therapeutic radiation techniques now rely on inhomogeneity corrected dose calculations.^(14)^ Convolution is, however, based on the physical densities of tissues obtained from tomographic studies and that can introduce errors to the calculation if artifacts are present. Potential inaccuracies arise mainly from external elements that change the apparent density of the tissues in the CT scan (i.e., contrast agent, intracranial clips, titanium cranioplasties, embolization materials) and the stereotactic frame itself. In our study, one of the targets (out of 85) was considerably affected by imaging artifact from the frame pins and, in this specific case, the uncertainty of the convolution algorithm was significantly high. A method of overriding the electron density in areas of artifact would significantly reduce this uncertainty.

A visual evaluation of the whole head CT scan should be sufficient to detect CT artifacts that potentially affect the convolution calculations. Adequate electron density calibration needs to be performed for each individual CT scan, and scanning protocol and special attention must be given to the consistency of the scanning procedure to reduce technical variability.

In our study the imaging procedure was done with the stereotactic frame and this can produce CT artifact itself, as seen in Fig. 2. Distortion from the frame ring occurs mainly in the lowest aspect of the scan and this is very unlikely to be part of a beam's incoming trajectory. However, areas of artifact caused by the fixation posts and pins are certainly likely to be crossed by the collimated beams. Apart from our incidental finding of a lesion located precisely under the fixation pin, it was not possible to investigate the effect of the frame artifact in the patients and their more complex radiosurgery plans. Nonetheless, our phantom studies demonstrated no change on treatment plans with the 8 and 16 mm collimators and only a minor change (4%, worst‐case scenario) if a single shot of the 4 mm collimator is used. This difference is probably undetectable for more complex multi‐isocenter treatment plans and the significance of this finding as a weakness of the convolution algorithm is debatable because the frame will also be in place at the time of treatment. No significant difference was noted between different pin materials.

Potential dosimetric inaccuracies have been suggested with onyx embolization material and the effect of other embolization agents has not been fully understood.^(16)^ In our study four AV M patients had undergone partial glue embolization, and the dose shift with the convolution algorithm in these cases was comparable to subjects who had not had endovascular treatment before GKR. These findings are consistent with the study by Mamalui‐Hunter et al.^(17)^ who concluded that dose reduction due to attenuation of the ^60^Co beam by the AV M embolization material was very small for glue (n‐ butyl 2 cyanoacrytate) and also for Onyx (ethylene vinyl alcohol) because of the high‐energy ^60^Co beam. No patient in our study had undergone embolization with Onyx and our findings of no increased uncertainty due to previous embolization apply only to glue embolizations.

### C. Head definition with CT

Implementation of the convolution algorithm also involves using CT outlining to define the shape of the head and this results in dosimetric differences of approximately 1.5%. The maximum dose discrepancy due to head shape approximation in our study was 3.4% and comparable results have been reported by similar studies.^18^, ^19^ Nakazawa et al.^(18)^ reported an average difference of ‐0.16% between measured and CT‐based contours with a maximum difference of 3.4% and concluded it was an acceptable range. The manual method is certainly a practical and convenient approach and the justification of a head CT scan for head definition only is debatable. However, the head is a complex irregular structure and CT outlining can generate a better 3D model. It should be used if a CT scan of the head is available for other clinical reasons or the convolution algorithm is to be used. The uncertainty from head approximation tends to be greater in deep‐seated targets and can be foreseen if significant discrepancies are noted on visual evaluation of the head contour.

## V. CONCLUSIONS

Inhomogeneity correction with the convolution algorithm results in a considerable, but consistent, dose shift compared to the TMR 10 algorithm traditionally used for GKR. No significant difference in relative dose distribution was noted and a reduction of the prescription dose may be necessary to obtain the same absolute dosimetric effect with the convolution algorithm. This study has revealed that dose to the cochlea during GKR is approximately 7% lower than initially predicted with the TMR 10 algorithm and further reduction may be achieved if prescription doses with the convolution algorithm are adjusted. Head shape definition using CT outlining can be used to reduce uncertainty from head shape approximations and CT artifact typically caused by the stereotactic frame, glue embolization material or different fixation pin materials do not systematically affect convolution calculations. Nonetheless, special attention must be given to cases with major CT artifacts around the target where the convolution algorithm may not optimally simulate dose distributions.

## ACKNOWLEDGMENTS

This study was funded by Queen Square Radiosurgery Centre.

## COPYRIGHT

This work is licensed under a Creative Commons Attribution 3.0 Unported License.
